# Skin Barrier Homeostasis in Atopic Dermatitis: Feedback Regulation of Kallikrein Activity

**DOI:** 10.1371/journal.pone.0019895

**Published:** 2011-05-25

**Authors:** Reiko J. Tanaka, Masahiro Ono, Heather A. Harrington

**Affiliations:** 1 Department of Bioengineering, Imperial College London, London, United Kingdom; 2 Immunobiology Unit, Institute of Child Health, University College London, London, United Kingdom; 3 Department of Dermatology, Graduate School of Medicine, Kyoto University, Kyoto, Japan; 4 Division of Molecular Biosciences, Imperial College London, London, United Kingdom; Rikagaku Kenkyūsho Center for Allergy and Immunology, Japan

## Abstract

Atopic dermatitis (AD) is a widely spread cutaneous chronic disease characterised by sensitive reactions (eg. eczema) to normally innocuous elements. Although relatively little is understood about its underlying mechanisms due to its complexity, skin barrier dysfunction has been recognised as a key factor in the development of AD. Skin barrier homeostasis requires tight control of the activity of proteases, called kallikreins (KLKs), whose activity is regulated by a complex network of protein interactions that remains poorly understood despite its pathological importance. Characteristic symptoms of AD include the outbreak of inflammation triggered by external (eg. mechanical and chemical) stimulus and the persistence and aggravation of inflammation even if the initial stimulus disappears. These characteristic symptoms, together with some experimental data, suggest the presence of positive feedback regulation for KLK activity by inflammatory signals. We developed simple mathematical models for the KLK activation system to study the effects of feedback loops and carried out bifurcation analysis to investigate the model behaviours corresponding to inflammation caused by external stimulus. The model analysis confirmed that the hypothesised core model mechanisms capture the essence of inflammation outbreak by a defective skin barrier. Our models predicted the outbreaks of inflammation at weaker stimulus and its longer persistence in AD patients compared to healthy control. We also proposed a novel quantitative indicator for inflammation level by applying principal component analysis to microarray data. The model analysis reproduced qualitative AD characteristics revealed by this indicator. Our results strongly implicate the presence and importance of feedback mechanisms in KLK activity regulation. We further proposed future experiments that may provide informative data to enhance the system-level understanding on the regulatory mechanisms of skin barrier in AD and healthy individuals.

## Introduction

Atopic dermatitis (AD) is a common chronic allergic skin disease characterised by dry, scaly skin, inflammation, increased skin permeability, susceptibility to allergy causing sensitive reactions to normally innocuous elements and vulnerability to surface infection [1], [2]. The lifetime prevalence of AD is estimated to 15–30% in children and 2–10% in adults while the incidence of AD has increased by 2- to 3-fold during the past 3 decades in industrialised countries [1], and thus AD has significant socioeconomic and personal impacts in these countries [3]. Recently, skin barrier dysfunction has been recognised as one of the key factors in the development of AD [2], [4], [5], although relatively little is understood about its underlying mechanisms due to its complexity.

**Figure 1:**
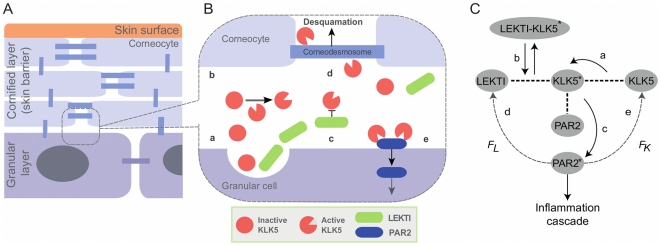
Models of skin desquamation. A: Cartoon model of skin desquamation. Skin barrier is physically composed of the cornified layer, where keratin-filled and anucleated keratinocytes (corneocytes) are densely packed with skin lipids. Corneocytes are interconnected by corneodesmosomes. Skin desquamation occurs by elimination of corneocytes at the skin surface. B: Cartoon model of protein interactions involved in KLK5 activation regulation. (a) KLK5 and their inhibitor LEKTI are secreted from granular cells into the intercellular space at the interface of cornified and granular layers; (b) KLK5 self-activates by proteolysis; (c) Direct binding of LEKTI inhibits the activity of KLK5; (d) Active KLK5 physically cleaves corneodesmosomes, which bind corneocytes together, resulting in elimination of corneocytes; (e) PAR2 is cleaved by active KLK5 to be activated and internalized. Figure was modified from [9]. C: Simplified model for KLK5 activation regulation system proposed in this paper. KLK5* and PAR2* represent the activated forms of KLK5 and PAR2, respectively. (a) KLK5 self-activation by proteolysis; (b) Association and dissociation of LEKTI and KLK5*; (c) PAR2 activation by KLK5*; (d) Feedback from PAR2* to production of LEKTI (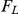); (e) Feedback from PAR2* to production of KLK5 (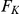); Inflammation level is denoted by the level of PAR2*.

The skin barrier is physically composed of the cornified layer, where keratin-filled and anucleated keratinocytes (corneocytes) are densely packed with skin lipids (Fig. 1A). Skin barrier homeostasis is attained by balancing the differentiation of granular layer keratinocytes to corneocytes against elimination of corneocytes at the skin surface (desquamation) [6]. The latter occurs as the result of cleavage of corneodesmosomes (Fig. 1B), which bind corneocytes together, by serine proteases called kallikreins (KLKs) [7]. Excessive activities of KLKs can impair the skin barrier via premature breakdown of corneodesmosomes by KLKs [8] and increase corneocyte desquamation. Accumulating evidence indicates malfunctions in the spatial and temporal control of KLK activity in AD patients is one of the main causes for their defective skin barrier homeostasis [9].

KLKs are synthesised as inactive precursors and are secreted into the extracellular space, where they are activated by another active KLK by irreversible proteolysis (Fig. 1B) [10]. The activity of each KLK is further regulated by direct interaction with proteinase inhibitors such as Lympho-epithelial Kazal-type related inhibitor (LEKTI) [11], and by changes in pH [12]. Indeed, compared to healthy control (HC), AD patients have the following three characteristics: (1) higher protein level of KLKs in stratum corneum [13], (2) significant decrease in the expression of *SPINK5* encoding LEKTI [14], and (3) higher pH level [15], [16], all of which result in higher KLK activity. In addition to KLKs and LEKTI, recent findings have suggested that protease-activated receptors type 2 (PAR2) plays a significant role in skin barrier homeostasis [17]–[19]. PAR2 is cleaved and activated by active KLKs, resulting in Ca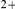 release and mitogen activated protein kinase (MAPK) activation. In physiological conditions (healthy status), PAR2 signalling is reported to regulate the differentiation of keratinocytes [20], while in pathological conditions including AD, this signalling upregulates cytokine production by keratinocytes and induces immune response [21].

The activities of KLKs and PAR2s are thus tightly regulated by a complex network of protein-protein interactions that remain, despite its pathological importance, poorly understood. Therefore, it is indispensable to reveal how different components such as KLK, LEKTI, PAR2 and pH affect the systems behaviour by their mutual interactions and feedback regulation, and to understand how these mechanisms are dysregulated at the system-level in AD patients. However, experimental data are currently limited and the entire regulatory mechanism is still obscure.

AD is a notoriously chronic disease: sensitive reactions including inflammation occur easily by external stimulus (eg. scratching) and may persist or even aggravate, even if the initiating stimulus no longer exists. These features, notably outbreak, persistence, and aggravation of inflammation, suggest the presence of a positive feedback loop [22], [23] in the regulatory system for KLK activity. Although such feedback loops have not been explicitly identified to date, feedback regulatory mechanisms of KLK activity are further suggested by the following experimental evidence: (1) cells within the inflammatory infiltrate produce KLKs as a product of the inflammatory response, in proportional level with the severity of a flare of AD [7], (2) both KLKs [13] and PAR2 [24] proteins are increased in AD lesions, (3) patients with different deficiency in *SPINK5* gene show different KLK expression level [25], (4) the kinetics of skin barrier recovery is accelerated in PAR2 knockout than wild-type [26], (5) keratinocytes have receptors for inflammatory cytokines such as IL1 and IL8 (downstream signals of PAR2 activation), thus can be activated in an autocrine manner [27], [28]. Importantly, KLK, LEKTI and PAR2 do not interact inside the cell, but are transported outside of the cell separately and only interact at the cell surface and in the extracellular space [9].

Accordingly, we conduct a systems-level investigation of the feedback regulation of KLK activity in skin. To achieve the overall aim of better understanding underlying mechanisms of skin barrier dysfunction in AD patients, we carry out the investigation in three steps. First, we develop a novel mathematical model of the KLK activation system and provide a framework to coherently explain the current experimental knowledge on AD. The mathematical model we propose in this paper consists of four core mechanisms for KLK activation: (1) KLK self-activation, (2) KLK inhibition by LEKTI, (3) PAR2 activation by KLK, and (4) feedback regulation of KLK and LEKTI via activated PAR2. The first three mechanisms have been rather well characterised experimentally while the feedback mechanism has been implicitly suggested based on different experimental evidence as described above [7], [13], [24]–[26] and awaits the explicit identification by experiments. Second, using this mathematical framework, we investigate the fundamental and core mechanisms responsible for qualitatively different behaviours of the system to characterise HC and AD patients. We hypothesise models with different feedback loops and identify the plausible system behaviours by bifurcation analysis. The proposed models successfully reproduce the clinically well-known and essential AD features: persistent inflammation triggered by lower level of external stimulus for AD than for HC. To gain further insight, we perform sensitivity analysis [29], motivating the detailed study of parameter-dependencies of system behaviours; furthermore, this analysis identifies the important balance between degradation rates and rates for feedback kinetics. Lastly, the model predictions are verified with experimental data. Since PAR2 activity is difficult to directly measure by conventional experiments including Western blotting of signalling proteins, we propose a novel way of evaluating PAR2 downstream signal activities using microarray analysis. Specifically, we apply principal component analysis (PCA) to microarray data of HC and AD samples and derive an indicator (PAR2 score) of the PAR2 downstream inflammation level that can capture the difference between HC and AD patients. The model predictions are then verified using the PAR2 score for HC and AD patients and confirm the coherency of the model. All the results presented here support the presence and significance of feedback loops in the regulation of KLK activity, and thus this work attempts to motivate a variety of future experiments for further study in order to better understand the fundamental regulatory mechanisms of skin barrier homeostasis in AD and healthy individuals.

## Results

### Construction of mathematical models: Model overview

We developed an ordinary differential equation model for regulation of KLK activation at the interface of cornified and granular layers in skin (Fig. 1C) consisting of three main mechanisms (dotted lines in Fig. 1C): (1) KLK self-activation, (2) KLK inhibition by LEKTI, and (3) PAR2 activation by active KLK, and hypothesised feedback loops (dotted arrows in Fig. 1C) from activated PAR2 to KLK and LEKTI production. This model focuses on KLK5, which is the primary KLK for skin desquamation and is involved in all four of the aforementioned mechanisms [30]. The complete model description is shown in the Methods section, together with the nominal parameter values in Table 1.

**Table 1:** Definitions of system parameters and values used in simulations.

Parameter	Description	Nominal value
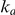	LEKTI-KLK5* association rate	1 (pH 4.5) [12]
		3 (pH 6.5) [12]
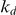	LEKTI-KLK5* dissociation rate	1 (pH 4.5) [12]
		2.5 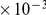 (pH 6.5) [12]
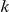	KLK5 activation rate	10 (pH 4.5) [50]
		50 (pH 6.5) [50]
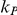	PAR2 activation rate	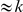 [50]
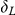	LEKTI degradation rate	0.5
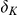	KLK5 degradation rate	1
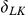	LEKTI-KLK5* degradation rate	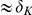
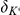	KLK5* degradation rate	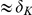
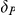	PAR2 degradation rate	0.5
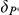	PAR2* degradation rate	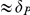
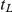	LEKTI production capability	1 (HC) [14]
		0.5 (AD-LEKTI) [14]
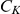	Half-saturation of KLK5 activation	50
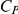	Half-saturation of PAR2 activation	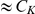
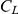	Inhibition constant for Model 2	5
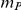	Basal production rate for PAR2	10
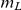	Basal production rate for LEKTI	1
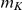	Basal production rate for KLK5	0
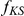	Rate of KLK5 production by stimulus	0.5
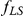	Rate of LEKTI production by stimulus	0.05 (Model 1)
		0 (Model 2)
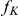	Feedback strength from PAR2* to KLK5	0–1
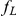	Feedback strength from PAR2* to LEKTI	0–0.5 (Model 1)
		0–10 (Model 2)

**Figure 2:**
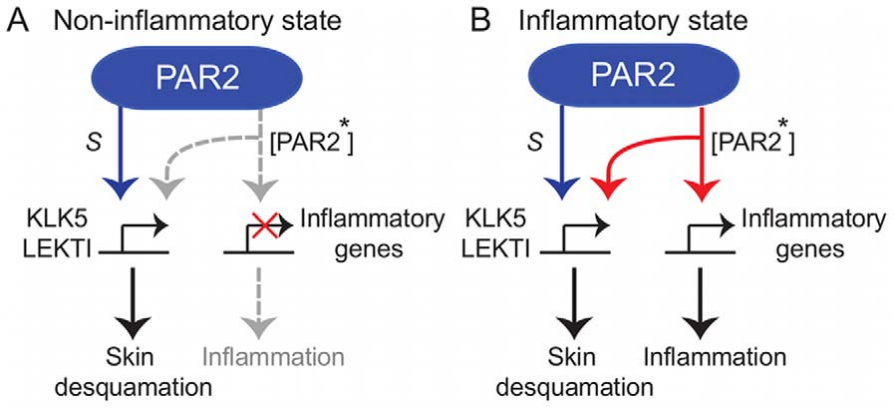
PAR2 signalling downstreams in granular cells. A: For the non-inflammatory states, a small amount of active PAR2, 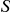, is constitutively produced, maintaining the basal activity of KLK5 production for the normal desquamation process. Various external stimuli can influence 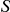 to be fluctuated around its nominal value. B: For the inflammatory states, a large amount of activated PAR2, [PAR2*], is induced and internalized, which then transduce stronger canonical signalling cascades and increase the expression of inflammatory genes including *IL1* 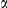, *IL1* 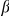, *IL8* and *TNF-* 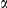.

In the first mechanism, KLK5 self-activation (Fig. 1C(a)), an inactive precursor form of KLK5 is cleaved to be an activated KLK5 (denoted by KLK5* hereafter) by itself. In the second mechanism, KLK5 inhibition (Fig. 1C(b)), LEKTI binds to and almost perfectly inactivates KLK5* [11] by producing the complex LEKTI-KLK5* that has no cleaving activity. In the next mechanism, PAR2 activation by KLK5* (Fig. 1C(c)), inactive PAR2 is cleaved and activated by KLK5*. For the non-inflammatory states, a small amount of active PAR2 (denoted 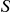) is constitutively produced, maintaining the basal activity of KLK5 production for the normal desquamation process (Fig. 2). Various external stimuli can influence 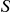 to be fluctuated around its nominal value. The integrated strength of external stimuli is accordingly represented by 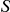. For the inflammatory states, a large amount of activated PAR2 (denoted by PAR2* hereafter) is induced and internalized, which then transduces stronger canonical signalling cascades and increases the expression of inflammatory genes including *IL1* 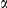, *IL1* 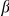, *IL8* and *TNF-* 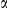. The inflammation level is accordingly represented by the level of PAR2* in our model.

Positive feedback loops from PAR2* to KLK5 production would reflect that KLK concentration increases when PAR2 is activated and inflammation occurs [7]. We investigated the possibilities of both positive (Model 1) and negative (Model 2) feedbacks from PAR2* to LEKTI production, as there is no strong experimental evidence to discriminate the most plausible feedback mechanism. The strength of the feedback from PAR2* to KLK5 and LEKTI are denoted by 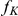 and 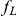 in our model, respectively. We assumed that PAR2 production is constant [31].

### Model behaviours: Outbreak and persistence of inflammation

**Figure 3:**
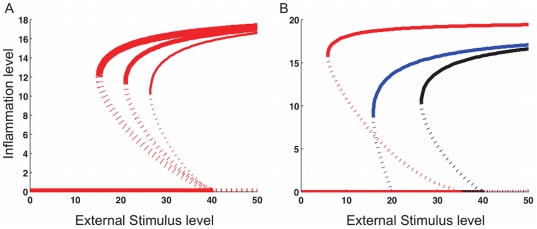
Bifurcation diagrams of Model 1 showing the inflammation outbreak and its persistence. Inflammation outbreak and its persistence appear as bistability of inflammation level as the external stimulus level changes. The solid and dotted lines show the stable and unstable steady states, respectively. A: The thickness of each bifurcation curve corresponds to positive feedback strength 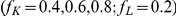. Stronger positive feedback leads to more persistent inflammation, as is shown by the larger range of the bistability. B: The behaviours are compared for HC (black), AD-LEKTI (blue), and AD-pH (red) with 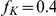 and 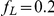. The inflammation threshold is lower for AD conditions than that for HC.

We first confirmed that the proposed model exhibits the expected characteristic behaviours of AD, that is, the flare of inflammation by external stimulus and its persistence despite a decrease of initial external stimulus (eg. scratching). We used bifurcation diagrams to delineate qualitatively different behaviour; they characterise the type of model behaviours in the presence of an external stimulus, similar to dose response curves in experimental biology which can be used to identify such changes in behaviour. As the external stimulus increases, the non-inflammatory (zero) state may change whereby there are two stable (bistable) steady states, corresponding to a high inflammatory and a non-inflammatory state. Bifurcation diagrams for Model 1 (Fig. 3A) illustrate stable and unstable steady states corresponding to solid and dotted lines, respectively. The thickness of each bifurcation curve corresponds to the strength, 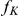, of the positive feedback to KLK5. Stronger positive feedback leads to more persistent inflammation, as shown by the larger range of the bistability. Persistent inflammation means that a significant decrease of external stimulus is required for the inflammation to cease. The inflammation level is also higher for the system with stronger feedback. The similar bifurcation behaviours are observed for Model 2 with negative feedback from PAR2* to LEKTI (supporting Fig. S1). The parameter dependency, especially to the feedback strength, of the bifurcations is investigated throughout this work.

**Figure 4:**
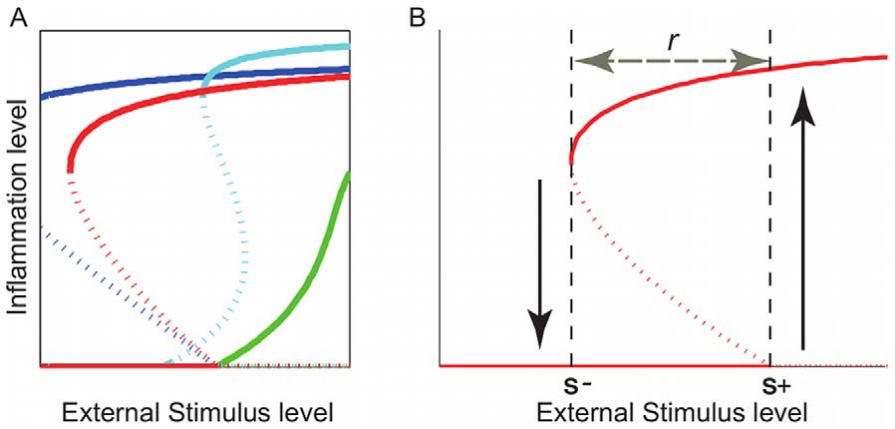
(A) Four bifurcation patterns exhibited by our models and (B) quantitative indices of bistable behaviours. A: Reversible bistability (red), irreversible bistability (blue), continuous monostability (green), and discontinuous monostability (cyan). Bistability patterns match the characteristic switching of inflammation. Irreversible bistability patterns correspond to more severe symptoms than reversible bistability patterns. B: Inflammation outbreaks at the inflammation threshold 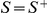 and persists until 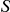 decreases to reach the deactivation threshold 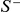, where the inflammation level returns to zero. The range of bistability 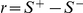 represents the required level of decrease in the external stimulus for the inflammation to cease. Smaller values of 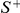 indicate an increased sensitivity of the skin to external stimulus; Larger values of 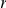 indicate that the inflammation is persistent.

The observed behaviours (Fig. 3A) are summarised in Fig. 4B: there is no inflammation (inflammation level is zero) at low external stimulus level 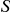; as 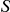 increases, inflammation outbreaks at a certain stimulus threshold for inflammation outbreak, 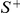, where there is a jump from no inflammation state to a higher inflammation state. Once inflammation occurs, the inflammation levels remain high even if 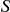 decreases, until 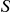 reaches the deactivation threshold of stimulus, 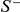, which is much smaller than the inflammation threshold 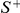. The hysteresis curve represents the persistence of inflammation despite the decrease of the external stimulus. At 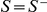, the inflammation level finally returns to zero, that is, the inflammation stops.

### AD characteristics: Smaller inflammation threshold for AD than for HC

We then verified that the proposed model captures characteristics of AD patients compared to HC. We characterised AD patients by the following two conditions: (1) limited LEKTI production rate and (2) high pH, based on the observation that LEKTI expression in AD is about 50% of that of HC [14] and AD skin generally exhibits higher pH (around 6.5) compared to HC (pH around 4.5), although there are large variances of the pH level for each individual and for different skin areas [15]. We compared the behaviours for the following three conditions: (1) limited LEKTI production rate at pH 4.5 (AD-LEKTI), (2) full LEKTI production rate at pH 6.5 (AD-pH), and (3) full LEKTI production rate at pH 4.5 (HC). AD-LEKTI condition is investigated by setting 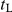, a parameter representing the LEKTI production rate in our model, to be half of the full LEKTI production rate. AD-pH condition is studied by changing pH-dependent parameters, 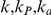, and 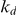 according to the literature (see Methods). We investigated AD-LEKTI and AD-pH conditions to clarify the respective effects of limited LEKTI production rate and high pH separately.

The bifurcation diagrams of Model 1 at different conditions clearly exhibit a lower inflammation threshold for both AD-LEKTI (blue) and AD-pH (red) conditions than that for HC (black), and a much higher inflammation level for AD-pH condition (Fig. 3B). This is consistent with the clinical features of AD which shows more sensitivity to external stimulus: low levels of external stimulus, which are innocuous to HC, can trigger inflammation in AD patients. Qualitatively similar behaviour was observed for Model 2 (Fig. S2). The lower inflammation threshold in AD conditions compared to HC is similarly observed regardless of feedback strength in both Models 1 and 2, while more detailed and quantitative comparison of behaviours for varied conditions with different feedback strength is shown in the next section.

### Classification of observed bifurcations

The bifurcation diagrams shown above exhibited bistability, indicating inflammation outbreaks by external stimuli; however, the proposed models can exhibit other behaviours for certain systems parameter combinations. Figure 4A demonstrates the four typical behaviours:

- Reversible bistability (red): Bistable states appear for 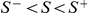 with the deactivation threshold 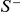 in the biologically feasible region of 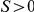. The inflammation is reversible, that is, it outbreaks at 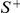 and ceases at 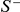;
- Irreversible bistability (blue): Bistable states appear for 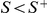. The inflammation is irreversible, that is, it persists even if 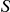 decreases to zero once it outbreaks at the threshold 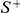, as 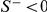 is not in the biologically feasible region of 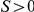.
- Continuous monostability (green): Monostable state appears for all 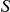. In this case, the inflammation occurs gradually as 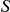 increases, which is contradictory to on/off switching of inflammation observed in AD symptoms;
- Discontinuous monostability (cyan): Monostable state appears for low and high 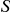 and there is some range of 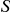 for which no stable points exist.

Among these four behaviours, only the reversible and irreversible bistability match the characteristic on/off switching of inflammation, while continuous and discontinuous monostability behaviours are not biologically plausible. Strictly speaking, 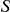 may not be decreased below a certain level in reality, and thus the reversibility of this model may not correspond to spontaneous regression of lesion in clinical circumstances. Therefore, the difference between reversible and irreversible bistability should be interpreted as an ordered scale for the disease severity: irreversible bistability corresponds to a more severe case. The systems that exhibit irreversible bistability and the ones of reversible bistability with small 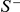 have to be modulated by changing some systems parameters to increase the deactivation threshold into the biologically feasible region, for example by topical cortiocosteroid, in order to stop the inflammation.

### Effects of feedback strength on model behaviours

**Figure 5:**
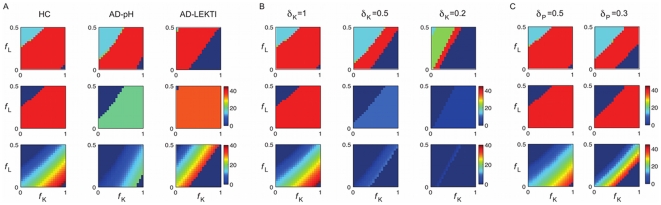
Bifurcations for Model 1 with different parameters. Calculated for 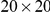 pair of feedback strength 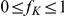 and 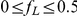. KLK production rate is higher at the bottom right corner. (Top) Bifurcation patterns with colours corresponding to those in Fig. 4A. Stronger KLK activation results in more severe symptoms of irreversible bistability. (Middle) Inflammation threshold 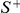 for bistability patterns; 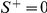 for monostability patterns. (Bottom) Range of bistability 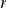 for reversible bistability; 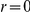 for other patterns. The inflammation is more persistent (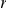 is larger) with stronger KLK activation. A: Comparison for HC, AD-LEKTI, and AD-pH. AD conditions exhibit smaller 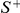 and more severe symptoms than HC. B: Comparison for different degradation rates for KLK5 and KLK5* in HC with 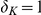 (nominal), 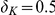 and 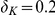. Slower KLK5 degradation (smaller 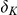) results in the stronger KLK activity and shows similar effects as in AD-pH condition leading to more irreversible bistability patterns and lower threshold values. C: Comparison for different degradation rates for PAR2 and PAR2* in HC with 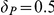 (nominal) and 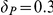. Slower PAR2 degradation (smaller 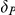) results in the stronger inflammation and shows similar effects as in AD-LEKTI condition leading to more irreversible bistability patterns and little changes in threshold values.

The four distinct bifurcation behaviours (Fig. 4A) are exhibited by Model 1 by varying feedback strength for different conditions (Fig. 5). We varied the feedback strength about 1000-fold and identified the range that exhibits the change of bifurcation patterns of our interest. Top rows in Fig. 5A show the patterns we observed for different pairs of 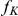 and 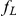, with the colours corresponding to those in Fig. 4A for different behaviours: reversible bistability (red), irreversible bistability (blue), continuous monostability (green), and discontinuous monostability (cyan). The patterns gradually change from discontinuous and continuous monostability at the top left corner to reversible bistability and then irreversible bistability at the lower right corner, where the KLK production rate is higher due to larger feedback strength to KLK 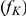 and the LEKTI production rate is lower due to smaller feedback to LEKTI 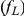. Stronger KLK activation thus results in more severe symptoms of irreversible bistability. The irreversible bistability behaviour is observed in larger parameter domains of the lower right corner for AD conditions. This result confirms that our model captures an aspect of the difference between HC and AD: the pair 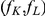 that results in the reversible bistability in HC leads to the irreversible bistability in AD conditions. That is, AD systems exhibit more severe symptoms than HC.

Bistable behaviours observed for varied feedback strengths and different conditions were investigated in more detail by calculating two quantitative indices (Fig. 4B) for bistable behaviours: (1) the inflammation threshold 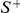 and (2) the range of bistability, 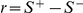, which indicates the necessary decrease of the external stimulus level for the inflammation to cease. Smaller values of 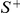 indicate an increased sensitivity of the skin to external stimulus; larger values of 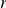 indicate that the inflammation is more persistent. Note, however, that 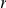 is only calculated for reversible bistability, not irreversible, as 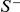 does not appear in the biologically feasible region for irreversible bistability.

Model 1 predicts much smaller 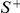 for AD conditions (middle rows in Fig. 5A) and large 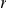 for stronger KLK activation at the bottom right corner of reversible bistability (bottom rows in Fig. 5A), as is consistent with the fact that the inflammation is more persistent with stronger KLK activation. Moreover, the stimulus has to decrease to almost zero for the inflammation to stop when KLK activation is strong, as shown by 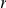 being almost equal to 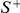. If KLK activation becomes stronger, the system exhibits irreversible bistability.

**Figure 6:**
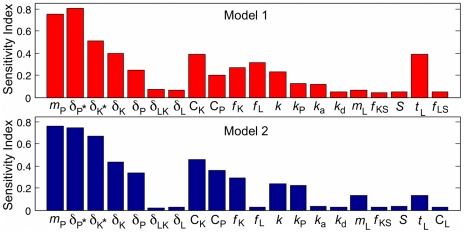
Sensitivity indicator for Models 1 and 2 calculated by eFAST. Global sensitivity analysis of Models 1 and 2 with respect to the steady state level of inflammation [PAR2*]. Baseline parameter values are given in Table 1. Parameters were perturbed over one order of magnitude (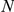 = 2000 simulations for eFAST).

The result of the same investigation for Model 2 (Fig. S3) exhibits similar qualitative features as those observed for Model 1. Note that KLK activation is stronger at the top right corner due to larger 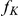 (resulting in more KLK production) and larger 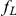 (resulting in less LEKTI production) in this model. Model 2 clearly demonstrates the transition of the bifurcation pattern from reversible to irreversible bistability as 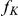 increases (Fig. S3A). The 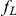-independence of the pattern is consistent with the result of the sensitivity analysis demonstrated in the next section (Fig. 6). Model 2 exhibits smaller 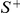 for AD conditions than for HC, similarly to Model 1, and also 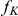-independence of 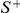 (Fig. S3B). Gradual increase of 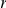 as the KLK activation increases (towards the top right corner of Fig. S3C) is also exhibited for Model 2, similarly for Model 1.

### Effects of degradation rates on model behaviours

In order to systematically investigate the effects of model parameters on system behaviours, we identified sensitive parameters for inflammation level [PAR2*] by performing global sensitivity analysis using eFAST [32], [33]. Both Models 1 and 2 exhibit high sensitivity to degradation rates of PAR2*, PAR2, KLK5*, and KLK5 (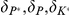, and 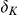), half-saturation of KLK5 and PAR2 activation (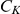 and 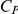), the feedback strength for KLK5 production 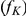, and pH-dependent activation rates for KLK5 and PAR2 (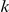 and 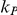) (Fig. 6). Model 1 is also sensitive to the feedback strength for LEKTI production (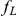) and LEKTI production rate 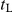. The sensitivity to PAR2 production rate 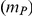 is also shown to be high; however, further study revealed 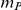 only affects the scaling of the inflammation level and not the bifurcations, and thus 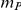-dependence was not investigated further. As the effects of 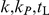 were discussed earlier, we systematically changed the remaining sensitive parameters together with the feedback strength to determine their type of effects on bifurcations.

We first studied the effects of KLK5 degradation rates (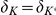) by changing 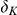 from 1 to 0.5 and 0.2, corresponding to the change of the half-life from approximately 15 to 30 and 70 minutes, respectively (Fig. 5B). Slower KLK5 degradation (smaller 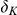) results in the stronger KLK5 activity, as KLK5 remains for a longer period of time, and thus shows similar effects as in AD-pH condition (Fig. 5A) leading to more irreversible bistability patterns and lower thresholds. The system with faster KLK5 degradation requires stronger (and thus faster) positive feedback strength to compensate the fast degradation in order to exhibit bistability. This result confirms the key role of the balance between the KLK5 degradation rates and feedback strength in determination of bifurcations. Similar effects were observed for half-saturation of KLK5 and PAR2 activation 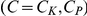 (Fig. S4), since smaller 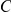 values also correspond to stronger activation.

We then investigated the effects of PAR2 degradation rates (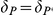) by changing 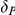 from 0.5 to 0.3, corresponding to the change of the half-life from approximately 30 to 60 minutes (Fig. 5C). Slower PAR2 degradation (smaller 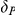) results in the stronger inflammation, as PAR2 stays for a longer period of time to initiate inflammation. Thus, we observed similar effects as in AD-LEKTI condition (Fig. 3A), in which KLK5* is less captured by LEKTI and activates more PAR2, resulting in more irreversible bistability patterns with little changes in thresholds.

Similar features were observed for Model 2: the effects of decreasing 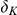 (Fig. S5) and of decreasing 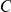 (Fig. S6) are similar to those of AD conditions (Fig. S3). The pattern of irreversible bistability becomes more relevant and the decrease of the threshold values are observed. The effects of decreasing 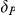 (Fig. S7) is also similar to AD-LEKTI condition, as it changes the bifurcation patterns but does not seem to significantly decrease the threshold.

### Characteristic features of AD and HC revealed by microarray data

The model predictions should be compared against experimental data that had not been used for the model construction. For this purpose, we analysed microarray data for AD and HC samples to derive their respective characteristic features to be tested with our models. While PAR2 activity, the key component in our model, is not directly measurable by conventional experiments, the downstream targets of PAR2 signalling are partially known to include *IL1*, *IL8* and other inflammatory genes, which can be measured by microarray analysis. Here we propose a reasonable indicator for the PAR2 activity by combining measurements of a set of PAR2 downstream genes.

A microarray dataset of AD patients and HC was obtained from the Gene Expression Omnibus (GEO) (accession number GSE5667) [34]. Using this dataset, we calculated the score (PAR2 score) which corresponds to the inflammation level in our model by weighting the expression data of selected possible PAR2 downstream genes (*ICAM1*, *IL8*, *TNF* 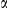, *CSF2*, *IL1* 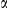, *IL1* 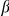, and *CCL17* [21], [35]). The weights were determined by the principal component score that represents the difference between AD and HC samples (see Methods). The original dataset for AD patients includes that for both lesional (LAD) and non-lesional (NLAD) skin area. LAD and NLAD were classified by the clinical features of the regional skin. In definition, LAD samples are obtained from eczematous skin regions with inflammation and NLAD samples are the ones with apparently normal skin without inflammation.

**Figure 7:**
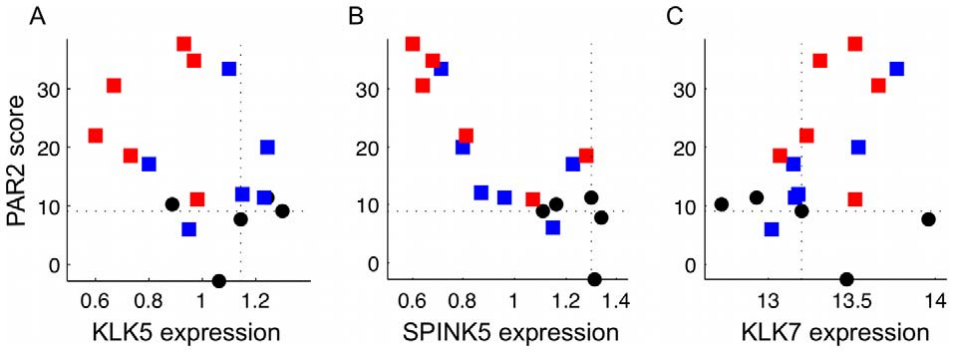
Microarray data for AD and HC samples. PAR2 score was derived using the data [34] of seven PAR2 downstream genes (see Methods). PAR2 score is plotted against expression data [34] of (A) *KLK5*, (B) *SPINK5*, and (C) *KLK7* for lesional AD (red squares), non-lesional AD (blue squares), and HC (black circles). The dotted lines indicate the median values of HC samples.

The calculated PAR2 score was plotted against the expression data of *KLK5*, *SPINK5* (encoding LEKTI), and *KLK7* in Fig. 7 for LAD (red squares), NLAD (blue squares), and HC (black circles). The dotted lines indicate the median of HC data that provides the reference value. Due to the small number of data and the individual variability, we are mostly concerned with whether the data value is high or low relative to the reference. Most of LAD and some of NLAD show high PAR2 score suggesting that inflammatory processes occur at certain degrees in these skin samples, whereas all of HC show low PAR2 scores, which reflect the absence of inflammation and are considered to be a background level in this analysis. This confirms that the PAR2 score calculated here is a reasonable indicator for the inflammation level.

Despite the small number of microarray samples, Fig. 7 exhibits clear relationships between PAR2 score and each expression level of *KLK5*, *SPINK5*, and *KLK7*. Data points with high PAR2 score show low *KLK5* expressions, although the point with the lowest *KLK5* expression level shows medium PAR2 score and thus no clear negative correlation was observed. The negative correlation between PAR2 score and *SPINK5* expression exhibited in Fig. 7B was consistently observed for different selections of possible PAR2 downstream genes (data not shown). A positive correlation between PAR2 score and *KLK7* expression is also observed, although ambiguous. Note that KLK7 is known to be involved in the skin desquamation process and activated by KLK5* while unable to activate PAR2. The dynamics of KLK7 thus cannot be captured in our model for KLK5 (Fig. 1C). The plots of PAR2 score and *KLK7* expression is shown here for a comparison with that of *KLK5*. Low expression of *SPINK5* in AD (Fig. 7B) is consistent with the data [14] showing lower LEKTI concentration in AD. Some NLAD samples even showed low *SPINK5* expression despite the low PAR2 score, which suggests that the low expressions of *SPINK5* are not dependent on the inflammation states of the skin.

The expressions of *KLK5* were low in AD samples, especially in those with high PAR2 score (Fig. 7A). It is not intuitively coherent to the previous data [13] that KLK5 protein concentration is higher in AD patients compared to HC. However, we note that the data is on the production level of KLK5 and not the concentration of KLK5 protein, and that *KLK5* mRNA (associated with the production rate) is suppressed while KLK5 protein level is high in the later phase of a time course experiment [36]. The positive correlation of *KLK7* expression level with PAR2 score (Fig. 7C) suggests that a different regulatory mechanisms is operating in KLK7. The qualitative difference of *KLK5* and *KLK7* expression data suggests that the PAR2 activation and the feedback via PAR2* is possibly an essential mechanism of KLK5 activation regulation.

### Model prediction captures characteristic features of AD revealed by microarray data

**Figure 8:**
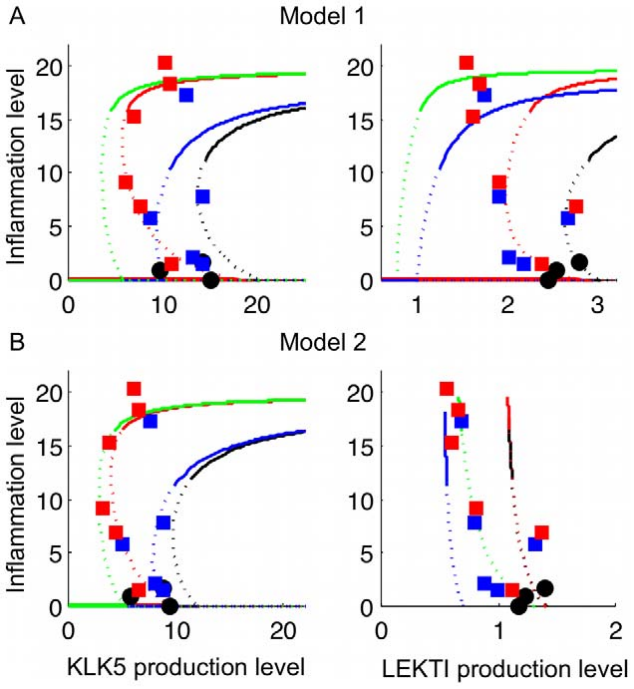
Model results of inflammation level against production level of KLK5 and LEKTI. A: Model 1 with 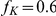 and 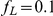, B: Model 2 with 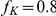 and 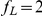. Lines with different colours correspond to different conditions: HC (black), AD-LEKTI (blue), AD-pH (red), and AD-LEKTI/pH (green). Microarray data in Fig. 7 is plotted for comparison after scaling: PAR2 score (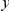) is scaled by 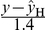, where 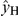 is the median of 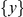 for HC, to compare with the inflammation level; *KLK5* (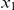) and *SPINK5* expression (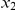) data are scaled by 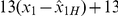 and 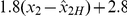 for comparison with Model 1, and 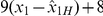 and 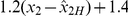 for comparison with Model 2, where 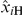 is the median of 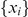 for HC. Data with positive values are only shown here.

The data showing the relationship between the expression levels of *KLK5* and *SPINK5* and the calculated PAR2 score (Figs. 7A and 7B) were then compared with corresponding simulation results for Models 1 and 2. In Fig. 8, the y-axis is the inflammation level ([PAR2*]) and the x-axis is KLK5 production level (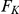) or LEKTI production level (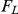) calculated with 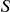 as a bifurcation parameter. As before, the solid and dotted lines correspond to the stable and unstable steady states, and the lines with different colours correspond to different conditions: HC (black), AD-LEKTI (blue), AD-pH (red) and AD-LEKTI/pH (green). AD-LEKTI/pH condition denotes the case with limited LEKTI production rate at pH 6.5, which is considered to reflect that of AD patients. PAR2 score and expression data were roughly scaled to facilitate the qualitative comparison of the data and the model results, revealing the qualitative characteristics of the data is coherently captured in the model results.

The observation of low *KLK5* expression level for LAD with high PAR2 score (Fig. 7A) were indeed remarkably reproduced by the model behaviours (Fig. 8). In both models, the inflammation level remains elevated even if the KLK5 production level decreases for AD-pH and AD-LEKTI/pH conditions, in the similar way as the inflammation level remains elevated despite a decrease in external stimulus level (Fig. 3). The qualitative characteristics of *KLK5* expression level could thus be captured by characteristic bistable behaviours exhibited by our models.

The negative correlation between PAR2 score and *SPINK5* expression (Fig. 7B) was also clearly reproduced by the model (Fig. 8). The production level of LEKTI (encoded by *SPINK5*) for Model 1 (Fig. 8A) predicts that the high inflammation level is achieved with low LEKTI production level for AD-LEKTI and AD-LEKTI/pH conditions. Similarly, under negative feedback (Model 2), low LEKTI production level occurs under AD-LEKTI and AD-LEKTI/pH conditions when the inflammation level is high; however, LEKTI production level for AD-LEKTI/pH conditions becomes as high as that for HC and AD-pH conditions when there is no inflammation (Fig. 8B).

While both the microarray data (Fig. 7A) and the model behaviours (Fig. 8) consistently showed low KLK5 production rate in AD especially at inflammatory states, the coherency of the model results with the previous data showing high KLK5 concentration for AD [13] was supported by our model results on the total concentration of all KLK5-related proteins (KLK5, KLK5*, and LEKTI-KLK5*), which ELISA [13] measures. Both Model 1 and Model 2 (Fig. S8) predict that the total KLK5 level is higher for higher inflammation states, which is consistent with the data in [13].

Accordingly, both the positive and negative feedback mechanisms (Model 1 and Model 2, respectively) could capture qualitative characteristics shown in the data. The combination of information revealed on KLK5 and LEKTI production and inflammation may suggest the main causes of the AD patients, either high pH or poor LEKTI production. Notably, Models 1 and 2 display obviously different behaviours for LEKTI production level vs inflammation level for all conditions. To distinguish between the two models, we require additional data, specifically uncovering information (such as pH or *SPINK5* mutation) for each sample, as described in the next section.

## Discussion

### Feedback regulation of KLK5 activity

The model development and analysis thus far confirmed that our hypothesised core model mechanisms captured the essence of inflammation outbreak by a defective skin barrier. Bifurcation analysis of the model with novel feedback mechanisms successfully exhibited AD characteristics: the more susceptibility to external stimulus, the stronger persistence of the inflammation, and the prevalence of more severe symptoms described by irreversible bistability for AD patients than for HC (Fig. 3B). While scattered experimental evidence indirectly suggested the presence of feedback mechanisms, there have been no previous attempts to develop mathematical models for KLK5 activation system related to skin barrier dysfunction. The coherency of our model results with the experimental data (Fig. 8), together with the qualitative difference we revealed in *KLK5* and *KLK7* expression data (Fig. 7), confirmed the feedback mechanisms via PAR2 activation to KLK5 and LEKTI production to be a key component in the model. Based on this theoretical result, the actual existence of the feedback from PAR2 to KLK5 and LEKTI should be experimentally verified by future experiments, such as those proposed in the next subsection.

Our model assumed the feedback from PAR2* to KLK5 to be positive, reflecting the increase of KLK5 level at the occurrence of the inflammation. The positive feedback from PAR2* to LEKTI (Model 1) allowed the system to finely determine the appropriate level of KLK activation by taking balance between two positive feedbacks for KLK and LEKTI; when inflammation occurs, both KLK5 and its inhibitor (LEKTI) are produced more to retain a homeostatic level of KLK5 activity. Another model considered assumed the negative feedback from PAR2* to LEKTI (Model 2). It resulted in acceleration of KLK5 activity and inflammation as KLK5 is produced more and its inhibitor (LEKTI) is produced less when inflammation occurs. Intuitively, the presence of such inherent positive acceleration is surprising: however, strong acceleration provides an explanation for strong and persistent inflammation in AD patients. The obvious difference of the results by Models 1 and 2 with regard to the negative correlation between PAR2 score and LEKTI production (Fig. 8) may prove useful to distinguish the two models as more data become available.

Coupled feedback loops, either dual-positive or combination of positive and negative loops are ubiquitously found in cellular systems [37], [38]. The combination of different kinematics, in addition to the positiveness and negativeness, of interconnected feedback loops have been found to play an important role to decide the system behaviour. For example, the combination of fast and slow (“dual-time”) positive feedback loops [37] and that of fast positive and slow negative feedback loops [38] make the system to be rapidly inducible and resistant to noise. The feedback strength in our model represent the rate of feedback kinetics: stronger feedback corresponds to faster feedback kinetics. Accordingly, it is intriguing to experimentally assess the feedback kinetics, as described below, to further our understanding of the regulatory mechanisms.

### Suggested experimental design for assessment of feedback kinetics

The feedback terms 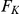 and 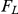 in our models are functions of both [PAR2*] and 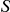. The feedback mechanisms involving PAR2* can be meaningfully assessed only through analysing the skin samples from AD patients, as (1) PAR2* is incorporated as a molecule that senses the inflammatory milieu in the skin barrier, and (2) inflammation does not occur without immunocytes. The other feedback through 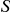, the basal activity of PAR2 corresponding to the external stimulus in our model, can be suitably analysed using organotypic culture of epidermis.

In the first system with clinical skin samples from AD patients, immunohistochemistry of skin biopsies from AD patients and HC using monoclonal antibodies (see below) against PAR2*, total PAR2, KLK5, and LEKTI would measure per cell concentrations of these proteins and provide information to deduce the feedback strength. Presumptive time course data of inflammation might be available by gathering the data of different skin lesions from an individual if they are described by dermatological terms such as erythema and plaque (roughly indicating early and chronic inflammation, respectively). The spacial information of protein concentration correlated with pathological features of the local area in the skin (e.g. spongiosis indicating a degree of inflammation) will be indispensable for future development of partial differential equation models. As a direct relationship with our current analysis, one can obtain a correlation between per cell protein concentrations and the bulk analysis of mRNA (Figs. 7 and 8) if each skin biopsy sample is analysed by both the immunohistochemistry and microarray.

The second system, organotypic culture of epidermis, is an experimentally feasible system for obtaining time course samples of basal PAR2 activation (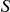), KLK5 production rate and concentrations of KLK5 proteins without the influence of other cells including immunocytes. Basal PAR2 level (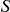) can be experimentally changed by mechanical stress and chemical stimulation. Time course samples of the organotypic culture would be analysed by real-time PCR of KLK5, LEKTI and PAR2 and by antibody-staining of anti-KLK5, anti-PAR2*, and total PAR2* for protein concentrations.

The two experimental systems described above require anti-total PAR2 and anti-active and/or phosphorylated PAR2 antibodies. It is important to generate monoclonal antibodies specific to the cleaved and/or phosphorylated form(s) of PAR2 protein [39] that can be used in both immunohistochemistry and Western blotting.

### Balance between degradation rates and feedback strength

Depending on the system parameters, our model exhibited four distinct behaviours in terms of bifurcation (Fig. 4A), among which reversible and irreversible bistability patterns were biologically plausible. We carried out the detailed study of parameter-dependencies of bifurcation to see their effects on bifurcation patterns, the inflammation threshold, and the range of bistability (Fig. 5). Our analysis revealed that balance among different parameters, especially that between KLK5 and PAR2 degradation rates and feedback strength was important to determine the bifurcations. Faster KLK5 and PAR2 degradation had to be compensated by faster (stronger) positive feedback to KLK5 for the system to exhibit biologically plausible bistability patterns. Slow degradations of KLK5 and PAR2 had the similar effects as AD-pH and AD-LEKTI conditions, respectively.

As a next step, it is critical to experimentally measure these key parameters, the degradation rates of KLK5 and PAR2. PAR2 protein is either membrane bound or in the cytoplasm and its degradation rate can be measured by the conventional degradation assay with protein synthesis inhibitor, cycloheximide. However, the degradation rate of KLK5 should be assessed in the extracellular space, especially in the skin barrier, which we modelled in this study. As it is still obscure which mechanisms degrade KLK5 in this space, the construction of *in vitro* degradation assay for KLK5 is difficult and impractical. Thus, the degradation rate is ideally measured using organotypic culture of epidermis, which is a culture system that allows full differentiation of keratinocytes with the layer structure as if like epidermis and has both the granular and cornified layer and therefore the skin barrier [40]. Time course analysis by immunohistochemistry with an appropriate data analysis of microscopic images would be the best way for measuring extracellular KLK5 in the granular and cornified layers.

### Microarray analysis of clinical samples

The model predictions were compared with the microarray data to confirm the feasibility of our proposed models. We used the microarray data for AD and HC samples to compare with the model results for the following two reasons. First, although KLK5 and LEKTI protein levels in the corneal layer can be measured, they may be different from the production rates by keratinocytes in the granular layer, which we modelled in this study. Thus, mRNA levels of these two genes may be one of the most reasonable and appropriate approximates of the production rates of these proteins. In fact, the protein expression levels of KLKs are increased when mRNA of the corresponding *KLKs* is increased, and mRNA and protein levels show significant correlations especially in the early phase of various kinds of stimulation [36]. Second, the same microarray analysis data allowed us to calculate PAR2 score, a novel indicator for PAR2 signalling. The PAR2 score, together with the expression data, clearly revealed characteristic features of AD and HC samples (Fig. 7), which were successfully reproduced by the model prediction (Fig. 8).

Our analysis suggests that microarray data can be more efficiently analysed if they are accompanied with detailed clinical features, which is rarely the case in published or publicly available data from microarray experiments. As we presented here, PAR2 score was calculated by the expression and annotation data (whether AD or HC) and was a useful indicator for assessing the activities of PAR2 and the degree of inflammatory reactions. If the current dataset was coupled with other annotation or clinical data such as skin pH and clinical features of eczema, some other scales could be made to analyse the correlations between the expression data and these clinical data (categorical data). For example, eczema may be described in various dermatological terms including erythema and plaque, which can be correlated with various conditions in our model. However, none of the currently available microarray dataset in dermatological research has full description of the skin lesion as far as the authors know. This is mainly because not many methods have been proposed for analysing both annotation and experimental data and because Minimum Information About a Microarray Experiment, a standard guideline for microarray experiments, does not sufficiently emphasize the importance of such annotation data. Microarray datasets with detailed clinical data would be undoubtedly informative for mathematical modelling studies.

### Possible further model development

There are several possible directions for further model development.

Firstly, our model focused on KLK5, the primary KLK for skin desquamation to capture the essence; however, KLKs constitute a family of 15 serine proteases (KLK1–KLK15) that activate each other. The network of KLK activation has been recently identified in vitro [41] and thus shall be included in the future model. Actually, AD is a paradigm case in which KLKs mediate pathogenesis, as KLKs have recently been recognised to play an important role in controlling both normal and pathological extracellular proteolysis and signalling [42]. Uncontrolled proteolytic activities of KLKs are associated with disease states including cancer, inflammation, and neurodegeneration, and they are overexpressed in various malignancies; for example, KLK3 expression is currently used as a marker for prostate cancer. Accordingly, our work has potential impacts on the research for these diverse diseases beyond AD and skin diseases.

Secondly, this model considered KLK5, LEKTI, and PAR2 as main proteins regulating the KLK5 activity and characterised AD patients by limited LEKTI production and high pH. However, there have been increasing number of studies showing that AD is associated with filaggrin deficiency, diminished level of skin lipids and increased epidermal proliferation and that the resulting congenital skin barrier defect is considered as the first trigger of the development of AD [43]. In this relation, there are two major consequences of the defective skin barrier that can be considered as AD characteristics. The first one is the persistent increase of percutaneous antigen penetration that could be avoided by the healthy skin barrier. Whereas our work focused on the persistent inflammation despite the decrease of initial external stimulus as a AD characteristic to be shown by our model, the stimulus level is actually difficult to be decreased for AD skin due to the persistent increase of antigen penetration. As a result, AD skin may suffer from much stronger persistence in inflammation. The effects of increased penetration of antigen, and of the resulting stronger stimulus, may be interpreted in our model analysis by rescaling the external stimulus level (abscissa) in the bifurcation diagrams or by increasing the feedback strengths 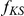 and 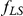 from 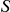 to KLK5 and LEKTI, respectively, for congenital defective skin barrier. Effects of these feedback strength can be investigated further in a similar way as in Fig. 5. While our proposed model focused on the inflammation level directly triggered by the external stimulus to elucidate the essential relationship between the external stimulus and inflammation, this increased stimulus level in AD skin shall be incorporated by adding extra feedback loops from PAR2* to 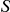 in the future model. The second AD characteristic resulting from the defective skin barrier is the systemic sensitisation, that is, a quicker and larger responses to antigens. It has been observed that an defective skin barrier produced by tape stripping gave rise to increased cell density of dendritic cells (e.g., Langerhans cells) [44], which play an important role in triggering a Th2 immune response and atopic inflammation. The sensitisation may be represented by the increased activation rates for KLK5 and PAR2 in our model, or investigated further by developing a systemic disease model, not a local model at the skin surface, that explicitly includes the immune cells and their interactions with keratinocytes.

The models developed in this paper were kept deliberately simple to capture the essence of the regulatory mechanisms for KLK5 activity. Since detailed information is still lacking, our model results are a first step to uncovering the mechanisms. Based on our findings, we invite experimentalists to perform the experiments, including those suggested above, to advance further model refinement and development. Ultimately this may provide a unified and quantitative basis for understanding possible causes of the disease, leading to diagnostic indices and pharmaceutical targets. Especially, persistent increase of percutaneous antigen penetration and systemic sensitisation mentioned above are two main features that shall be considered in the future models to entangle the complex interactions among genetic and environmental factors, the skin barrier, and immune deficiencies that lead to AD manifestations.

### Concluding remarks

This paper investigated feedback regulatory mechanisms for skin barrier homeostasis through combination of (1) model development based on recent experimental findings, (2) application of sensitivity analysis and bifurcation analysis, and (3) model validation using microarray data. To fill the gap between the model studies and experimental studies, we proposed a novel indicator for inflammation level, which is the key component of our model but is difficult to be measured by conventional experimental methods, by applying PCA to microarray data and also suggested future experiments in detail that would enhance further development of the model. AD is such a complex disease that has not been fully captured by any experimental systems, and its fundamental understanding will be extensively enhanced by a systems-level investigation of the KLK5 activation mechanism using model analysis like the one proposed here. This work is, to the best of the authors' knowledge, the first study for modelling KLK5 activation system that is responsible for skin barrier homeostasis, providing a framework to coherently understand the current experimental knowledge on AD.

## Methods

### Model description

Our model (Fig. 1C) is described as
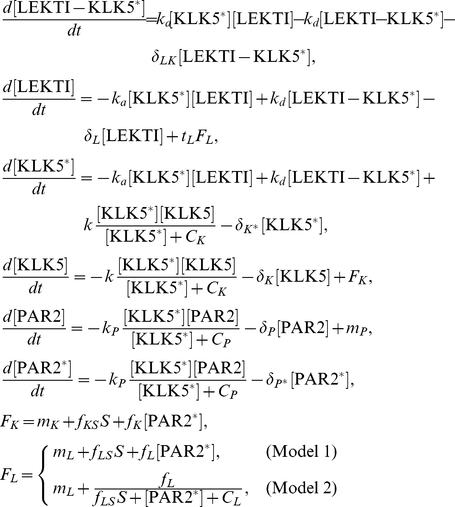
where 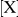 indicates the concentration of X and 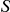 represents the external stimulus level, i.e. basal activation level of PAR2 when there is no inflammation (Fig. 2). KLK5 and PAR2 activations are described by Michaelis-Menten type equations as they are bounded by the availability of KLK5 and PAR2, respectively. The input functions 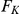 and 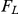 correspond to production processes of KLK5 and LEKTI proteins, respectively. We consider two models, Model 1 and Model 2, which have positive and negative feedback from PAR2* to LEKTI, respectively. We non-dimensionalised the ODE model described above using the values 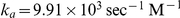 and 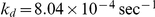 obtained for pH 4.5 [12] in order to minimise the effects of parameter uncertainties due to the lack of knowledge in system parameters. All the other parameters are defined relative to these numbers.

### Parameter estimation

The system parameters in the model are summarised in Table 1 with their nominal values used for the simulation and sources for each parameter estimate. Although estimates for some parameters are not available and there is no way at present to extract these numbers experimentally due to the difficulty in experiments, it is still possible to derive meaningful conclusion by numerical simulation and analysis of the model without them; especially our model analysis aimed to capture qualitative, and not quantitative, difference of HC and AD patients. Indeed, the lack of parameter estimates motivates several specific experiments to measure them, as proposed in the Discussion.

We investigated about 1000-fold range for each parameter to identify the parameter range to exhibit bifurcation of our interest, carried out global sensitivity analysis to identify sensitive parameters, and studied the effects of changes in these sensitive parameters on system behaviours. The activation rates (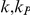) and half-saturation (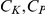) for activation of KLK5 and PAR2 were assumed to be same, as both KLK5 and PAR2 are activated by KLK5* and there is no quantitative information available for their difference. The degradation rates were assumed to be same for active and inactive forms of KLK5 (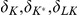) and PAR2 (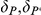) due to the lack of knowledge. Protease (KLK5) degradation rate (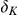) was considered to be larger compared to those for LEKTI (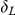) and PAR2 (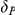). Basal production rate for KLK5 (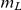) was assumed to be 0 as it only shifts the KLK5 production rate and does not affect the dynamics (data not shown). Rate of LEKTI production by stimulus for Model 2 (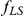) was assumed to be 0 as the qualitative features discussed in this paper were not affected by this parameter (data not shown).

### Model analysis

We solved 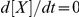 to determine the steady state concentrations for each model species in terms of the parameters. Since only one analytically tractable steady state was present, corresponding to the zero 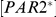 (non-inflammatory) state, the steady state solutions were numerically calculated for the specific model and its proposed conditions at fixed parameter values. The number of steady states varied from one to three depending on the parameter values.

In order to determine which parameters were responsible for the number of fixed points and its corresponding stability, global sensitivity analysis of Models 1 and 2 were performed using eFAST. Parameters were perturbed over one order of magnitude (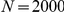 simulations). The calculated sensitivity indices motivated our selection of bifurcation parameters and numerical bifurcation analysis was conducted.

To further investigate the behaviour of the stability of the system while multiple parameters were varied, we constructed the characteristic polynomial of the Jacobian evaluated at each steady state (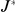) and determined the stability of each steady state using the Routh-Hurwitz criterion, a preferred method for nonlinear ODE systems, which uses the coefficients of 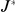 and hence bypasses solving the roots of each polynomial [45], [46]. We performed this analysis for both Models using the nominal parameter values (Table 1) unless otherwise stated while also varying three parameters: stimulus level (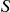), feedback strength from PAR2* to KLK5 (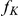) and that to LEKTI (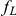), to explore the behaviours of inflammation level, 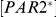. Specifically, this stability analysis enabled us to classify the monostable, bistable and irreversible bistable 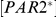 behaviours, ranges of bistability and threshold value 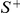 governing the switch from a low to high 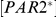 state.

### Derivation of PAR2 score

PAR2 activity, the key component in our model, is not directly measurable, since we cannot employ the common methods for measuring signalling activities, such as phosphorylation of signalling protein by Western blotting or measurements of Ca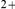 influx, due to the fact that the downstream signals of PAR2 include MAPK and Ca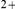 release, both of which are not specific to PAR2. However, the downstream targets of PAR2 signalling are partially known and include *IL1*, *IL8* and other inflammatory genes, which we can measure by microarray analysis.

Microarray analysis has a unique feature that it measures ten of thousands of genes at the same time and now widely used in experimental medicine, while it is difficult to obtain many samples because of the high costs. A reasonable indicator for the target signal may be obtained by combining measurements of a set of the related genes, although there has not been any established methods to determine the appropriate weights for the measurement of each gene transcript. Here we used principal component analysis (PCA) to determine the reasonable weights based on our recent findings (paper submitted by Ono *et al.*) that PCA decomposes the variances between microarray samples and that the method is further refined by employing a bootstrapping technique. The PAR2 score derived in the way described below by using PC scores as weights could successfully reflect the difference between AD and HC groups.

Let 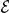 be a microarray expression data, where the 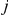-th column (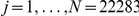) represents an expression level of the 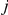-th probe with the zero mean and the 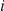-th row (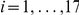) corresponds to the 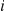-th individual (either AD patients or HC). PCA applied to 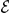 provides the PC score 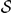 of the PC axis that represents the difference between AD patients and HC. We used a bootstrapping technique to get the PC score in a stable manner as follows. At the 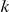-th bootstrap repetition (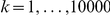), 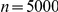 out of 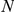 probes on the affymetrix microarray *hgu133a* are randomly resampled, and a new matrix 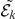 is obtained using both the resampled probes and the 13 probes corresponding to 7 selected PAR2 downstream genes (*ICAM1*, *IL8*, *TNF* 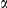, *CSF2*, *IL1* 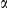, *IL1* 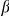, and *CCL17*). PCA applied to 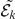 provides the PC score 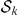. Subsequently, we obtain a PAR2 score 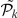 for the 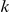-th bootstrap repetition by 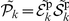, where 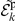 and 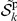 are submatrices of 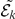 and 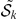, respectively, corresponding to the probes for the PAR2 downstream genes. The distribution of the weights 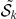 (Fig. S9) indicated that *CCL17* (207900_at) has the largest contribution to the PAR2 score as a single probe, while *IL-8* contributes almost equivalently to the PAR2 score with its two probes 202859_x_at and 211506_s_at. The PAR2 score 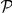 is obtained by taking the average of 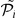. Thus, 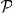 represents the presumptive effects of PAR2 downstream genes in terms of the disease activity of AD in each individual.

### Softwares

Oscill8 [47] were used for numerical bifurcation analyses. Calculations of Routh-Hurwitz stability criterion were performed using Maple 13 (Maplesoft, Waterloo, Ontario, Canada). Microarray data were normalised by *mas5* of the *affy* package of Bioconductor [48]. All figures were created using MATLAB version R2009b (The MathWorks, Inc., Natick, MA, USA). MATLAB add on toolboxes used include SBTOOLBOX2 [49] for global sensitivity analysis and SBML export.

## Supporting Information

Figure S1
**Bifurcation diagram of Model 2 showing the inflammation outbreak and its persistence.** The solid and dotted lines show the stable and unstable steady states, respectively. The thickness of each bifurcation curve corresponds to positive feedback strength 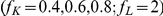. Stronger positive feedback leads to more persistent inflammation, as is shown by the larger range of the bistability.(TIF)

Figure S2
**Bifurcation behaviours of Model 2 for HC and AD conditions.** The behaviours are compared for HC (black), AD-LEKTI (blue), and AD-pH (red) with 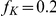 and 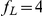. The inflammation threshold is lower for AD conditions than that for HC.(TIF)

Figure S3
**Bifurcations for Model 2 with different feedback strength.** Calculated for 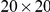 pairs of feedback strength in the range of 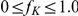 and 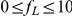 for HC, AD-pH, and AD-LEKTI. A: Bifurcation patterns with colours corresponding to those in Fig. 4A. B: Inflammation threshold 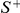. C: Range of bistability 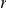 for reversible bistability.(TIF)

Figure S4
**Bifurcations for Model 1 with different half-saturation** 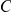 **for PAR2 and KLK5 activation.** Calculated for 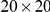 pair of feedback strength 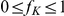 and 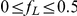 for 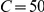 (nominal), 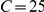 and 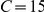. A: Bifurcation patterns with colours corresponding to those in Fig. 4A. B: Inflammation threshold 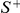 for bistability patterns; 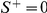 for monostability patterns. C: Range of bistability 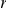 for reversible bistability; 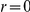 for other patterns.(TIF)

Figure S5
Bifurcations for Model 2 with different degradation rates for KLK5 and KLK5*. Calculated for 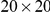 pairs of feedback strength in the range of 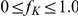 and 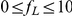 for 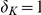 (nominal), 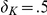 and 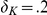. A: Bifurcation patterns with colours corresponding to those in Fig. 4A. B: Inflammation threshold 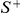. C: Range of bistability 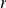 for reversible bistability.(TIF)

Figure S6
**Bifurcations for Model 2 with different half-saturation** 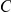 **for PAR2 and KLK5 activation.** Calculated for 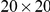 pairs of feedback strength in the range of 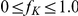 and 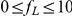 for 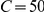 (nominal), 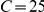 and 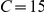. A: Bifurcation patterns with colours corresponding to those in Fig. 4A. B: Inflammation threshold 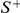 for bistability patterns; 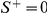 for monostability patterns. C: Range of bistability 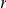 for reversible bistability; 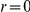 for other patterns.(TIF)

Figure S7
Bifurcations for Model 2 with different degradation rates for PAR2 and PAR2*. Calculated for 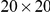 pair of feedback strength 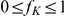 and 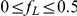 for 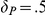 (nominal) and 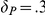. A: Bifurcation patterns with colours corresponding to those in Fig. 4A. B: Inflammation threshold 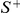 for bistability patterns; 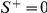 for monostability patterns. C: Range of bistability 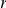 for reversible bistability; 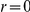 for other patterns.(TIF)

Figure S8
**Model results of total KLK level for HC and AD conditions.** Total KLK5 include KLK5, KLK5* and LEKTI-KLK5*. The behaviours are compared for HC (black), AD-LEKTI(blue), and AD-pH (red) Total KLK level is larger when the external stimulus level is higher. A: Model 1 with 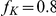 and 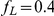. B: Model 2 with 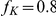 and 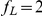.(TIF)

Figure S9
**Calculated weights of PAR2 downstream genes for PAR2 score.** Weights for 13 probes corresponding to the seven PAR2 downstream genes (*ICAM1*, *IL8*, *TNF* 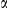, *CSF2*, *IL1* 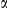, *IL1* 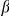, and *CCL17*) were obtained by applying PCA to microarray data with 10000 bootstrap repetition. Plots show the median (red bar), 25–75th percentile (box plot), non-outlier range (whiskers) and outliers (red cross) for each probe. ICAM(a): 202637_s_at *ICAM1*, ICAM(b): 202638_s_at *ICAM1*, ICAM(c): 215845_s_at *ICAM1*, IL8(a): 202859_x_at *IL8*, IL8(b): 211506_s_at *IL8*, TNFa: 207113_s_at *TNF* 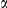, CSF(a): 210228_at *CSF2*, CSF(b): 210229_s_at *CSF2*, IL1a(a): 208200_at *IL1* 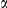, IL1a(b): 210118_s_at *IL1* 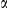, IL1b(a): 205067_at *IL1* 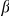, IL1b(b): 39402_at *IL1* 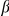, CCL17: 207900_at *CCL17*.(TIF)

## References

[1] Bieber T (2010). Atopic dermatitis.. Ann Dermatol.

[2] Callard RE, Harper JI (2007). The skin barrier, atopic dermatitis and allergy: a role for langerhans cells?. Trends Immunol.

[3] Mancini AJCS, Kaulback K (2008). The socioeconomic impact of atopic dermatitis in the united states: a systematic review.. Pediatr Dermatol.

[4] Cork MJ, Danby SG, Vasilopoulos Y, Hadgraft J, Lane ME (2009). Epidermal barrier dysfunction in atopic dermatitis.. J Invest Dermatol.

[5] Eichenfield LF, Hanifin JM, Luger TA, Stevens SR, Pride HB (2003). Consensus conference on pediatric atopic dermatitis.. J Am Acad Dermatol.

[6] Milstone LM (2004). Epidermal desquamation.. J Dermatol Sci.

[7] Cork MJ, Robinson DA, Vasilopoulos Y, Ferguson A, Moustafa M (2006). New perspectives on epidermal barrier dysfunction in atopic dermatitis: Gene-environment interactions.. J Allergy Clin Immunol.

[8] Voegeli R, Rawlings AV, Breternitz M, Doppler S, Schreier T (2009). Increased stratum corneum serine protease activity in acute eczematous atopic skin.. Br J Dermatol.

[9] Ovaere P, Lippens S, Vandenabeele P, Declercq W (2009). The emerging roles of serine protease cascades in the epidermis.. Trends Biochem Sci.

[10] Eissa A, Diamandis EP (2008). Human tissue kallikrein as promiscuous modulators of homeostatic skin barrier functions.. Biol Chem.

[11] Borgono CA, Michael IP, Komatsu N, Jayakumar A, Kapadia R (2007). A potential role for multiple tissue kallikrein serine proteases in epidermal desquamation.. J Biol Chem.

[12] Deraison C, Bonnart C, Lopez F, Besson C, Robinson R (2007). Lekti fragments specifically inhibit klk5, klk7, and klk14 and control desquamation through a ph-dependent interaction.. Mol Biol Cell.

[13] Komatsu N, Saijoh K, Kuk C, Liu AC, Khan S (2007). Human tissue kallikrein expression in the stratum corneum and serum of atopid dermatitis patients.. Exper Dermatol.

[14] Roedl D, Traidl-Hoffmann C, Ring J, Behrendt H, Braun-Falco M (2009). Serine protease inhibitor lymphoepithelial kazal type-related inhibitor tends to be decreased in atopic dermatitis.. J Euro Acad Dermatol Venereol.

[15] Choi SJ, Song MG, Sung WT, Lee DY, Lee JH (2003). Comparison of transepidermal water loss, capacitance and ph values in the skin between intrinsic and extrinsic atopic dermatitis patients.. J Korean Med Sci.

[16] Sparavigna A, Setaro M, Gualandri V (1999). Cutaneous ph in children affected by atopic dermatitis and in healthy children: a multicenter study.. Skin Res Technol.

[17] Dery O, Corvera CU, Steinhoff M, Bunnett NW (1998). Proteinase-activated receptors: novel mechanisms of signaling by serine proteases.. Am J Physiol Cell Physiol.

[18] Rattenholl A, Steinhoff M (2003). Role of proteinase-activated receptors in cutaneous biology and disease.. Drug development research.

[19] Steinhoff M, Buddenkotte J, Shpacovitch V, Rattenholl A, Moormann C (2005). Proteinaseactivated receptors: transducers of proteinase-mediated signaling in inammation and immune response.. Endocrine Reviews.

[20] Demerjian M, Hachem JP, Tschachler E, Denecker G, Declercq W (2008). Acute modulations in permeability barrier function regulate epidermal cornification: role of caspase-14 and the protease-activated receptor type 2.. Am J Pathol.

[21] Briot A, Deraison C, Lacroix M, Bonnart C, Robin A (2009). Kallikrein 5 induces atopic dermatitis-like lesions through par2-mediated thymic stromal lymphopoietin expression in netherton syndrome.. J Exp Med.

[22] Ferrell JE (2002). Self-perpetuating states in signal transduction: positive feedback, doublenegative feedback and bistability.. Current Opinion in Chemical Biology.

[23] Mitrophanov AY, Groisman EA (2008). Positive feedback in cellular control systems.. BioEssays.

[24] Buddenkotte J, Stroh C, Engels IH, Moormann C, Shpacovitch VM (2005). Agonists of proteinase-activated receptor-2 stimulate upregulation of intercellular cell adhesion molecule-1 in primary human keratinocytes via activation of nf-kappa b.. J Invest Dermatol.

[25] Komatsu N, Saijoh K, Juyakumar A, Clayman GL, Tohyama M (2008). Correlation between spink5 gene mutations and clinical manifestations in netherton syndrome patients.. J Invest Dermatol.

[26] Hachem JP, Houben E, Crumrine D, Man MQ, Schurer N (2006). Serine protease signaling of epidermal permeability barrier homeostasis.. J Invest Dermatol.

[27] Schulz B, Michel G, Wagner S, Suss R, Beetz A (1993). Increased expression of epidermal il-8 receptor in psoriasis. down-regulation by fk-506 in vitro.. J Immunol.

[28] Groves R, Giri J, Sims J, Dower S, Kupper T (1995). Inducible expression of type 2 il-1 receptors by cultured human keratinocytes. implications for il-1-mediated processes in epidermis.. J Immunol.

[29] Saltelli A, Ratto M, Tarantola S, Campolongo F (2005). Sensitivity analysis for chemical models.. Chem Rev.

[30] Emami N, Diamandis EP (2008). Human kallikrein-related peptidase 14 (klk14) is a new activator component of the klk proteolytic cascade.. J Biol Chem.

[31] Roosterman D, Schmidlin F, Bunnett NW (2003). Rab5a and rab11a mediate agonist-induced traffcking of protease-activated receptor 2.. Am J Physiol Cell Physiol.

[32] Marino S, Hogue IB, Ray CJ, Kirschner DE (2008). A methodology for performing global uncertainty and sensitivity analysis in systems biology.. J Theor Biol.

[33] Chu Y, Jayaraman A, Hahn J (2007). Parameter sensitivity analysis of il-6 signalling pathways.. IET Syst Biol.

[34] Plager DA, Leontovich AA, Henke SA, Davis MD, McEvoy MT (2007). Early cutaneous gene transcription changes in adult atopic dermatitis and potential clinical implications.. Exp Dermatol.

[35] Yoo J, Omori M, Gyarmati D, Zhou B, Aye T (2005). Spontaneous atopic dermatitis in mice expressing an inducible thymic stromal lymphopoietin transgene specifically in the skin.. J Exp Med.

[36] Morizane S, Yamasaki K, Kabigting FD, Gallo RL (2010). Kallikrein expression and cathelicidin processing are independently controlled in keratinocytes by calcium, vitamin d3, and retinoic acid.. J Invest Dermatol.

[37] Brandman O, Ferrell JE, Li R, Meyer T (2005). Interlinked fast and slow positive feedback loops drive reliable cell decisions.. Science.

[38] Kim D, Kwon YK, Cho KH (2006). Coupled positive and negative feedback circuits form an essential building block of cellular signaling pathways.. Bio Essays.

[39] Ricks TKTJ (2009). Phosphorylation of protease-activated receptor-2 differentially regulates desensitization and internalization.. J Biol Chem.

[40] O'Shaughnessy RFHJ, Choudhary I (2010). Interleukin-1 alpha blockade prevents hyperkeratosis in an in vitro model of lamellar ichthyosis.. Hum Mol Genet.

[41] Yoon H, Laxmikanthan G, Lee J, Blaber SI, Rodriguez A (2007). Activation profiles and regulatory cascades of the human kallikrein-related peptidases.. Journal of biological chemistry.

[42] Sotiropoulou G, Pampalakis G, Diamandis EP (2009). Functional roles of human kallikrein-related peptidases.. J Biological Chemistry.

[43] Proksch E, Folster-Holst R, Brautigam M, Sepehrmanesh M, Pfeiffer S (2009). Role of the epidermal barrier in atopic dermatitis.. J German Soc Dermat.

[44] Proksch E, Brasch J, Sterry W (1996). Integrity of the permeability barrier regulates epidermal langerhans cell density.. Br J Dermatol.

[45] Barnett S, Šiljak DD (1977). Routh's algorithm: A centennial survey.. SIAM Review.

[46] Eissing T, Conzelmann H, Gilles ED, Allgöwer F, Bullinger E (2004). Bistability analyses of a caspase activation model for receptor-induced apoptosis.. J Biol Chem.

[47] Conrad E (2005). Oscill8 documentation: Introduction.. http://oscill8.sourceforge.net/doc/.

[48] Gentleman RC, Carey VJ, Bates DM, Bolstad B, Dettling M (2004). Bioconductor: open software development for computational biology and bioinformatics.. Genome Biology.

[49] Schmidt H, Jirstrand M (2006). Systems biology toolbox for matlab: a computational platform for research in systems biology.. Bioinformatics.

[50] Brattsand M, Stefansson K, Lundh C, Haasum Y, Egelrud T (2005). A proteolytic cascade of kallikreins in the stratum corneum.. J Invest Dermatol.

