# Supplementary figures and images for: Skin Barrier Homeostasis in Atopic Dermatitis: Feedback Regulation of Kallikrein Activity
Source: PLoS One. 2011 May 25;6(5):e19895. doi: 10.1371/journal.pone.0019895 (PMC3102059; doi:10.1371/journal.pone.0019895)

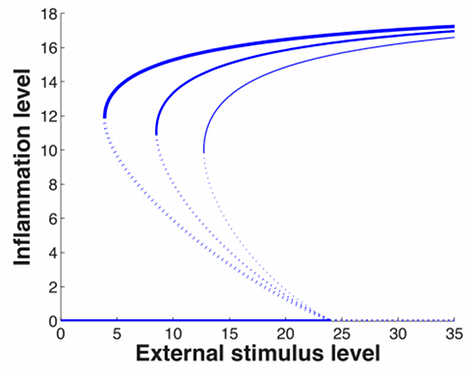

Supplement: Figure S1 — Bifurcation diagram of Model 2 showing the inflammation outbreak and its persistence. The solid and dotted lines show the stable and unstable steady states, respectively. The thickness of each bifurcation curve corresponds to positive feedback strength . Stronger positive feedback leads to more persistent inflammation, as is shown by the larger range of the bistability. (TIF) [file pone.0019895.s001.tif]

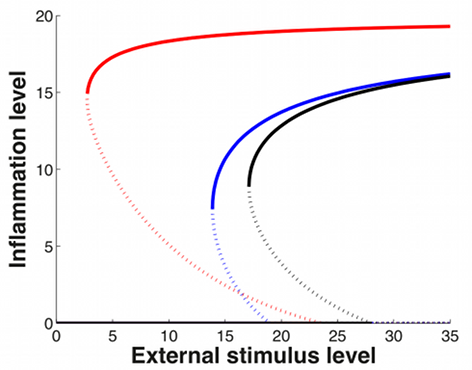

Supplement: Figure S2 — Bifurcation behaviours of Model 2 for HC and AD conditions. The behaviours are compared for HC (black), AD-LEKTI (blue), and AD-pH (red) with and . The inflammation threshold is lower for AD conditions than that for HC. (TIF) [file pone.0019895.s002.tif]

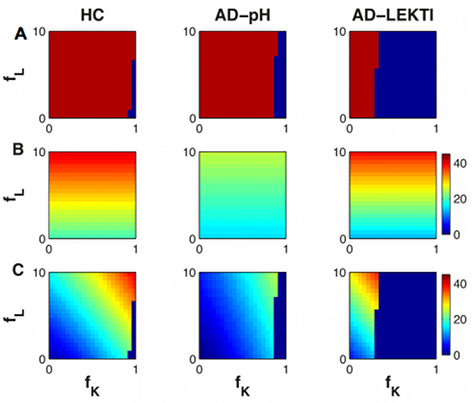

Supplement: Figure S3 — Bifurcations for Model 2 with different feedback strength. Calculated for pairs of feedback strength in the range of and for HC, AD-pH, and AD-LEKTI. A: Bifurcation patterns with colours corresponding to those in Fig. 4A. B: Inflammation threshold . C: Range of bistability for reversible bistability. (TIF) [file pone.0019895.s003.tif]

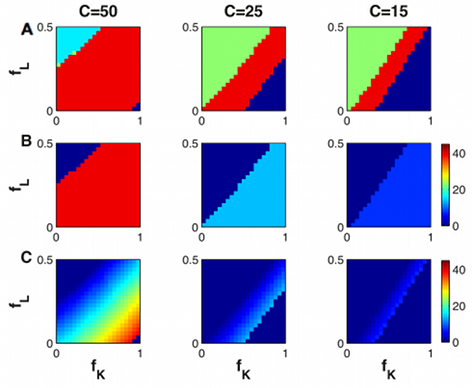

Supplement: Figure S4 — Bifurcations for Model 1 with different half-saturation for PAR2 and KLK5 activation. Calculated for pair of feedback strength and for (nominal), and . A: Bifurcation patterns with colours corresponding to those in Fig. 4A. B: Inflammation threshold for bistability patterns; for monostability patterns. C: Range of bistability for reversible bistability; for other patterns. (TIF) [file pone.0019895.s004.tif]

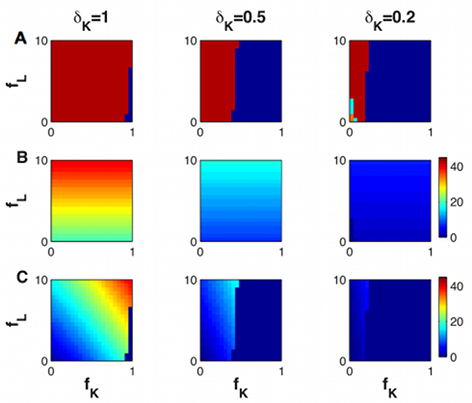

Supplement: Figure S5 — Bifurcations for Model 2 with different degradation rates for KLK5 and KLK5*. Calculated for pairs of feedback strength in the range of and for (nominal), and . A: Bifurcation patterns with colours corresponding to those in Fig. 4A. B: Inflammation threshold . C: Range of bistability for reversible bistability. (TIF) [file pone.0019895.s005.tif]

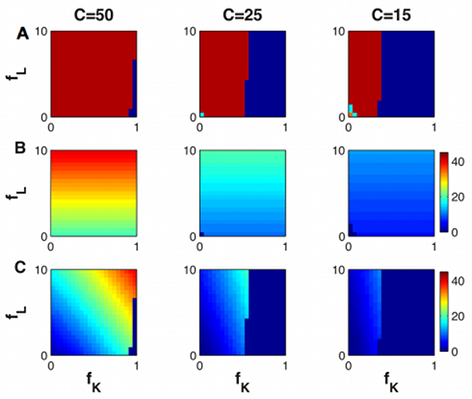

Supplement: Figure S6 — Bifurcations for Model 2 with different half-saturation for PAR2 and KLK5 activation. Calculated for pairs of feedback strength in the range of and for (nominal), and . A: Bifurcation patterns with colours corresponding to those in Fig. 4A. B: Inflammation threshold for bistability patterns; for monostability patterns. C: Range of bistability for reversible bistability; for other patterns. (TIF) [file pone.0019895.s006.tif]

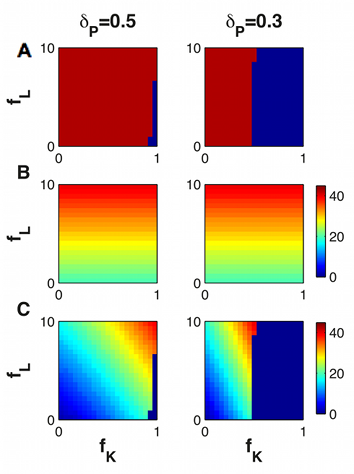

Supplement: Figure S7 — Bifurcations for Model 2 with different degradation rates for PAR2 and PAR2*. Calculated for pair of feedback strength and for (nominal) and . A: Bifurcation patterns with colours corresponding to those in Fig. 4A. B: Inflammation threshold for bistability patterns; for monostability patterns. C: Range of bistability for reversible bistability; for other patterns. (TIF) [file pone.0019895.s007.tif]

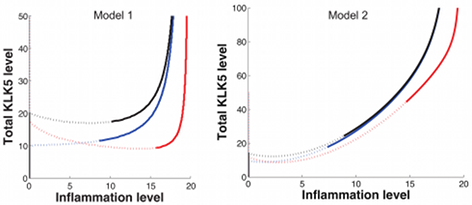

Supplement: Figure S8 — Model results of total KLK level for HC and AD conditions. Total KLK5 include KLK5, KLK5* and LEKTI-KLK5*. The behaviours are compared for HC (black), AD-LEKTI(blue), and AD-pH (red) Total KLK level is larger when the external stimulus level is higher. A: Model 1 with and . B: Model 2 with and . (TIF) [file pone.0019895.s008.tif]

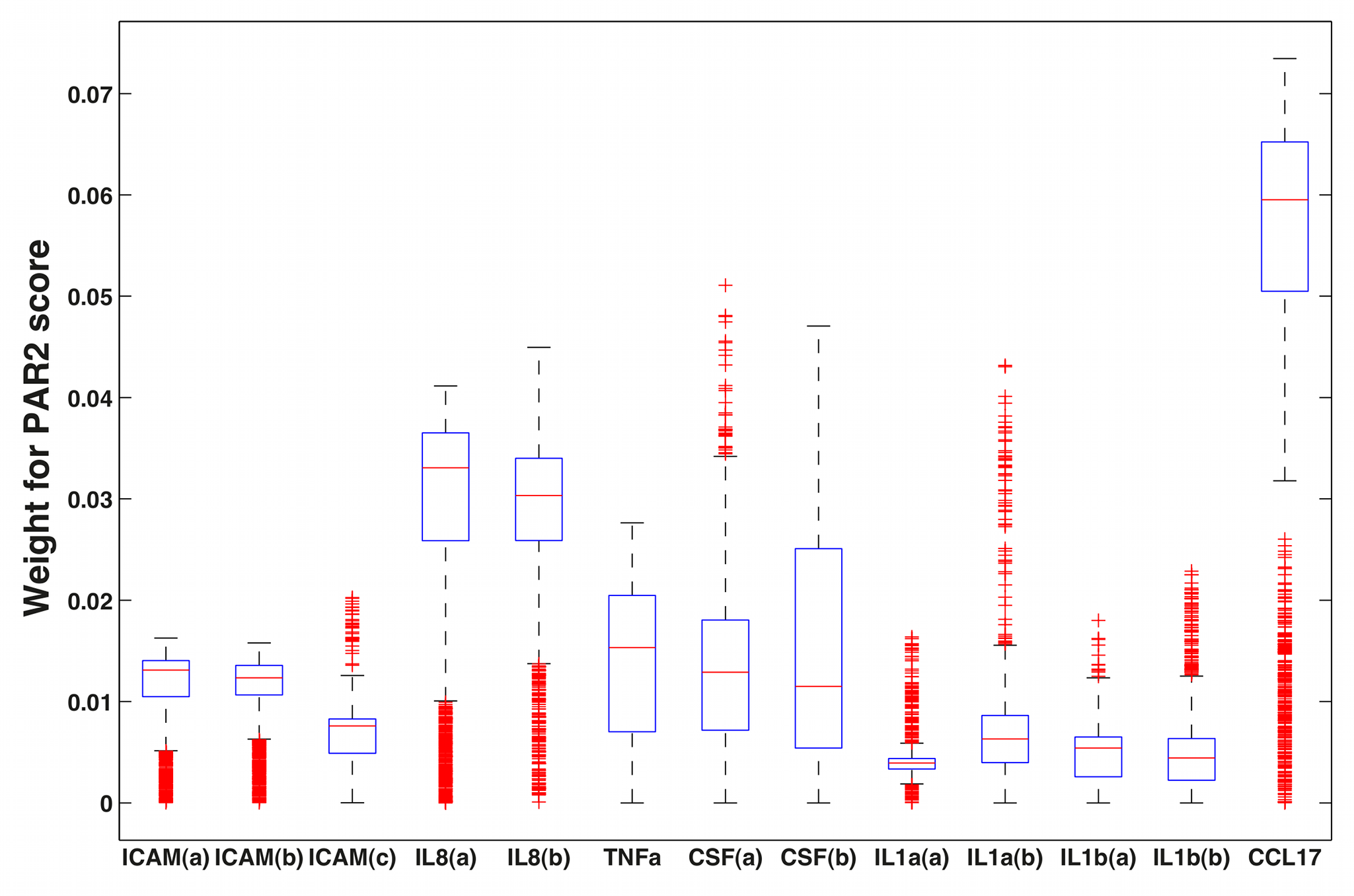

Supplement: Figure S9 — Calculated weights of PAR2 downstream genes for PAR2 score. Weights for 13 probes corresponding to the seven PAR2 downstream genes (ICAM1, IL8, TNF , CSF2, IL1 , IL1 , and CCL17) were obtained by applying PCA to microarray data with 10000 bootstrap repetition. Plots show the median (red bar), 25–75th percentile (box plot), non-outlier range (whiskers) and outliers (red cross) for each probe. ICAM(a): 202637_s_at ICAM1, ICAM(b): 202638_s_at ICAM1, ICAM(c): 215845_s_at ICAM1, IL8(a): 202859_x_at IL8, IL8(b): 211506_s_at IL8, TNFa: 207113_s_at TNF , CSF(a): 210228_at CSF2, CSF(b): 210229_s_at CSF2, IL1a(a): 208200_at IL1 , IL1a(b): 210118_s_at IL1 , IL1b(a): 205067_at IL1 , IL1b(b): 39402_at IL1 , CCL17: 207900_at CCL17. (TIF) [file pone.0019895.s009.tif]
